# Diagnostic efficacy of multiple MRI parameters in differentiating benign vs. malignant thyroid nodules

**DOI:** 10.1186/s12880-018-0294-0

**Published:** 2018-12-03

**Authors:** Hao Wang, Ran Wei, Weiyan Liu, Yongqi Chen, Bin Song

**Affiliations:** 1 Department of Radiology, Minhang Branch, Zhongshan Hospital, Fudan University, Shanghai, China; 2 Department of General Surgery, Minhang Branch, Zhongshan Hospital, Fudan University, Shanghai, China; 3 Department of Pathology, Minhang Branch, Zhongshan Hospital, Fudan University, Shanghai, China

**Keywords:** Diffusion weighted imaging, Magnetic resonance imaging, Thyroid carcinoma, Thyroid nodule

## Abstract

**Background:**

Diffusion weighted imaging (DWI) has a good diagnostic value for malignant thyroid nodules, but the published protocols suffer from flaws and focus on the apparent diffusion coefficient (ADC). This study investigated the diagnostic performance of multiple MRI parameters in differentiating malignant from benign thyroid nodules.

**Methods:**

This was a retrospective study of 181 consecutive patients (148 benign and 111 malignant nodules, confirmed by pathological results). The patients underwent conventional MRI, DWI, and dynamic contrast-enhanced MRI before surgery. The chi-square test and the Student t test were used to compare the conventional features and ADC value between malignant and benign groups. Multivariate logistic regression was used to identify the independent predictors and to construct a model. Receiver operator characteristic (ROC) curve analysis was used to assess the diagnostic performance of the independent variables and model.

**Results:**

Tumor diameter, ADC value, cystic degeneration, pseudocapsule sign, high signal cystic area on T1-weighted imaging, ring sign in the delayed phase, and irregular shape showed significant differences between two groups (all *P* < 0.05). The multivariable analysis revealed that ADC value (OR = 694.006, *P* < 0.001), irregular shape (OR = 32.798, *P* < 0.001), ring sign in the delayed phase (OR = 20.381, *P* = 0.004), and cystic degeneration (OR = 8.468, *P* = 0.016) were independent predictors. Among them, ADC performed the best in discriminating benign from malignant nodules, with an area under the curve (AUC) of 0.95, 0.90 sensitivity, and 0.91 specificity. When the independent factors were combined, the diagnostic performance was improved with an AUC of 0.99, 0.97 sensitivity, and 0.95 specificity.

**Conclusions:**

ADC value could discriminate between benign and malignant thyroid nodules with a good performance. Subjective features such as the ring sign, irregular shape, and cystic degeneration associated with malignant thyroid nodules could provide complementary information for differentiation.

## Background

About, 19–67% of the healthy, asymptomatic individuals are diagnosed with nodules of the thyroid [1]. Malignant thyroid nodules account for 5–15% of all thyroid nodules [2, 3]. With the rapid economic development of China, healthcare access and screening are improving in China and the detection and prevalence of thyroid nodules is reaching epidemics proportions, from 8% in 2002 to 25% in 2013 [4], as has been observed in developed countries [5].

Although ultrasound (US) is widely used to detect thyroid nodules and determine their malignant potential, the US features and index of each grade of the Thyroid Imaging Reporting and Data System (TI-RADS) remain controversial [6, 7]. US-guided fine-needle aspiration biopsy (FNAB) is widely used for the diagnosis of thyroid nodules [7]. Nevertheless, up to 7% of the nodules yield non-diagnostic cytology and an additional 15–30% of fine-needle aspiration cytology (FNAC) show an indeterminate cytology [7, 8].

DWI reflects the random Brownian motion of water molecules within a voxel of tissue. The movement of the tissue water molecules between the two opposing gradients will result in dephasing, which is seen as signal loss. ADC can be quantified calculated with repeating the sequence in different magnetic field strengths [9]. The ADC depends largely on the presence of barriers to diffusion within the water microenvironment, namely, cell membranes and macromolecules [10]. Malignant thyroid nodules usually have a lower ADC value compared to benign nodules [11–15], but this is controversial [16] and most of these studies used relatively low b values (< 500 × 10^− 3^/m^2^) [11, 14]. DWI has been shown to be of diagnostic value for Graves’ Disease [17], but DWI studies of thyroid nodules are relatively rare because the head and neck region is very heterogeneous and contains a variety of tissues that include fat, muscle, air, soft tissues, glands, and bones. In addition, MRI is more expensive and less available than US. Nevertheless, US has 40–80% sensitivity and 40–96% specificity for malignant thyroid nodules, highlighting the need for complementary examinations. In addition, previous DWI studies of thyroid nodules focused on the ADC [11, 12, 14, 16, 18].

Therefore, in the present study, we sought to improve the image quality with a special coil on the neck surface and a breath-hold technique, and to investigate the value of multiple MRI parameters for the diagnosis of thyroid carcinoma. Quantitative and qualitative parameters were examined.

## Methods

### Patients

This was a retrospective diagnostic study. Between January 2013 and December 2016, 479 consecutive patients underwent thyroidectomy at our hospital. Among them, 254 patients had undergone MRI examination within one week before surgery at the Department of Radiology. This study was approved by the institutional review board of our hospital. The requirement for individual written informed consent was waived.

The inclusion criteria were: 1) thyroid nodule that underwent surgery; and 2) available MRI data obtained within one week of surgery. The exclusion criteria were: 1) lesion size < 3 mm by US (*n* = 39); 2) poor image quality deemed to be non-diagnostic after review (*n* = 15); or 3) lesions with complete cystic changes (*n* = 19).

Among the 181 patients included, 140 patients underwent subtotal thyroidectomy, 25 underwent lobectomy, and 16 underwent total thyroidectomy.

### Multiparametric MRI protocol

MRI was performed with a 1.5 T scanner (EXCITE HD GE Healthcare, Waukesha, WI, USA) equipped with an 8-channel special neck surface coil (Chenguang Medical Technology Ltd., Shanghai, China). All patients were examined using the same machine, coil, and scan series.

The MR imaging protocol included T1-weighted image(T1WI), T2-weighted image(T2WI), DWI, and contrast enhanced T1WI of thyroid. Coronal fat-suppressed T2WI (repetition time (TR)/ echo time (TE), 1280 ms/85 ms; slice thickness; 4 mm; gap, 1 mm; matrix, 288 × 192; number of excitations (NEX), 4; field of view (FOV), 18 cm); axial T1WI (TR/TE, 460 ms/8 ms; slice thickness, 4 mm; gap, 0.5 mm; NEX, 2; FOV, 14 cm; matrix, 288 × 192); axial fat-suppressed T2WI (TR/TE, 3000 ms/85 ms, slice thickness, 4 mm; gap, 0.5 mm; NEX, 4;FOV, 14 cm; matrix, 320 × 224). DWI was performed using a single-shot echo planar imaging sequence with the diffusion gradient b factor = 800 s/mm^2^. Imaging parameters for DWI were: TR/TE, 6550 ms/minimum; FOV, 14 cm; NEX, 4; matrix, 128 × 128; slice thickness, 4 mm; and gap = 0.5 mm. Contrast enhancement studies were implemented using axial T1WI obtained with a fast-spoiled gradient recalled echo (TR/TE, 5.7 ms/1.7 ms; FOV, 14 cm; matrix, 192 × 256; NEX, 1). Gadolinium (Magnevist, Bayer HealthCare Pharmaceuticals, Montville, NJ, USA) was intravenously injected at 0.2 ml/kg body weight and 3 ml/s, followed by a 20 ml saline flush. In each patient, a phase was performed prior to the injection of contrast medium. Six phases were obtained after injection of the contrast agent at 30, 60, 120, 180, 240, and 300 s. In the contrast-enhanced protocol, breath-hold was performed during each phase. Spatial saturation bands were also used to remove signal from overlying fat and adjacent tissues. All patients received training on the breath-hold technique before the MRI examination.

### Image analysis

Two radiologists (13 and 18 years of MRI experience) reviewed all images using an AW4.5 workstation (GE Healthcare, Waukesha, WI, USA). Each radiologist was blinded to histopathology results. All images were reviewed independently by the two radiologists. Discrepancies were solved by discussion.

Lesions were evaluated for location, size (the largest linear dimension of nodules), shape (regular or irregular), margin (clear or vague), signal intensity heterogeneity (homogeneous or heterogeneous on T2WI), cystic degeneration, high signal cystic area on T1WI, ADC value of solid tumor area, and degree and pattern of enhancement. ROI (region of interest) of solid tumor area were manually drawn in the largest slice of lesions on ADC maps. The solid tumor areas were defined as a bright signal intensity identified on DWI images and dark signal intensity on ADC maps. Meanwhile, obvious areas of cystic changes, hemorrhage, calcification, and lesion margins need to avoid based on combined T2WI, contrast enhanced T1WI and DWI. The size of ROIs was determined to correspond with the darkest portion of lesions on ADC maps ranged 10-50 mm^2^. The ADC values were measured twice and then the average value was taken.

In this study, blood vessels, thyroid tissue, and muscles were used to assess subjectively the degree of early enhancement of the nodule, which was categorized as: mild (the enhancement was similar to that of adjacent muscle tissues); moderate (the enhancement was higher than that of adjacent muscle tissues but lower than that of blood vessels); and marked (the enhancement approached that of blood vessels). The pattern of enhancement reflected the dynamic contrast enhancement of thyroid nodules after the injection of the contrast agent in the delayed phase with ‘wash in’ or ‘wash out’. The ring sign was defined as the central area of the nodule showing ‘wash out’, whereas the peripheral area showed persistent enhancement. The pseudocapsule sign was defined as the tumor area showing a clear capsule after contrast agent administration in the delayed phase.

### Statistical analysis

Categorical data were presented as percentage and analyzed using the chi-square test. Continuous data were tested using the Kolmogorov-Smirnov test. Normally distributed continuous data were presented as means ± standard deviation and analyzed using the Student t test. Non-normally distributed data were presented as medians (range). All statistical analyses were performed with SPSS 23 (IBM, Armonk, NY, USA). Two-sided *P*-values < 0.05 were considered statistically significant. Binary logistic regression was used to identify the features that were independently predictive of malignant thyroid tumor. Variables demonstrating a significant association with malignant thyroid lesions were entered into the model in a forward stepwise method. The final model was selected based on variables with *P* < 0.05. The odds ratios (OR) and 95% confidence intervals (95%CI) were used as a measure of the relative magnitude of an association between predictor variables and malignant tumor. ROC curves were used to determine the cutoff value to differentiate the parameters of malignant from benign tumors.

## Results

### Characteristics of the patients

**Fig. 1:**
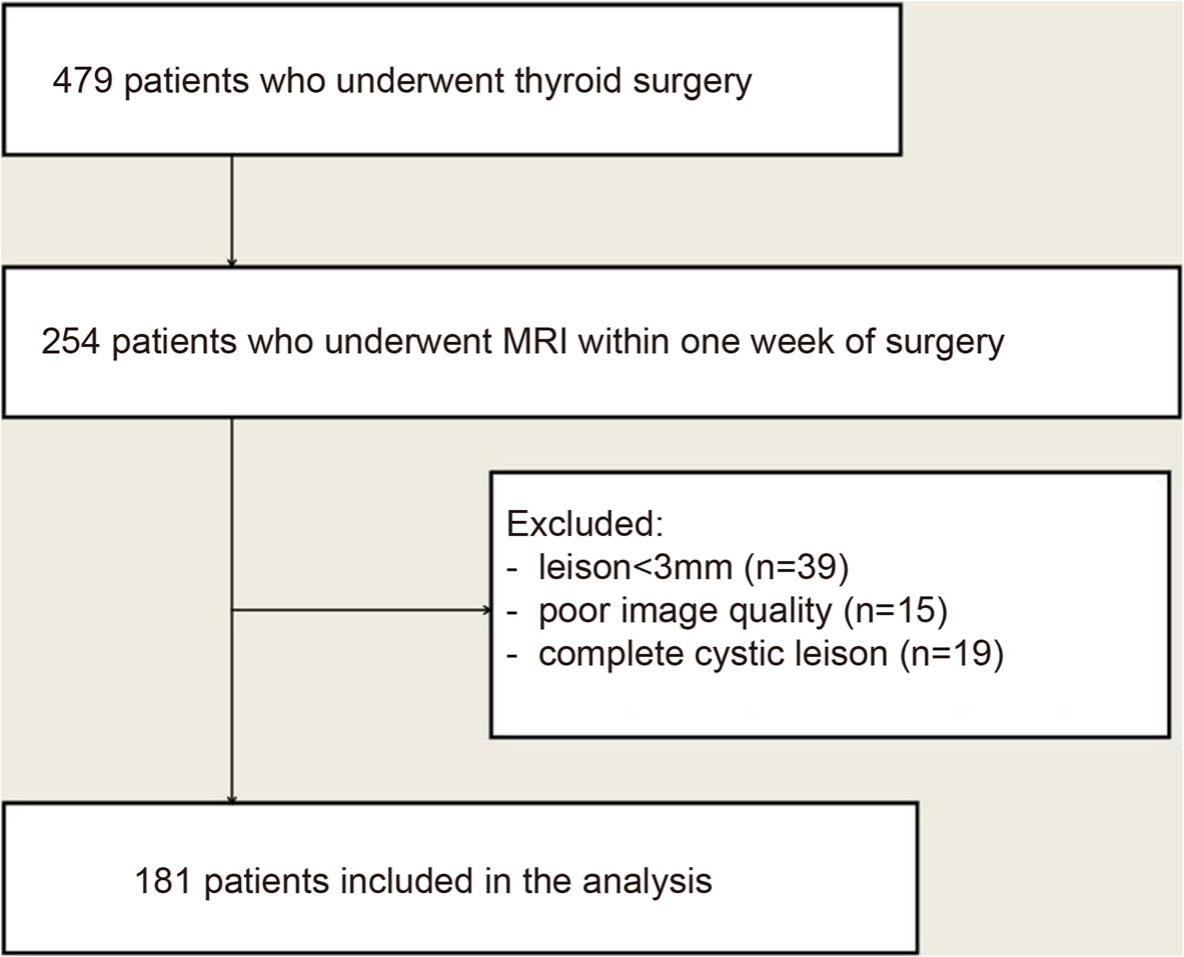
Patient flowchart

Figure 1 presents the patient flowchart. From 479 patients who underwent thyroid surgery, 254 patients underwent MRI examination within 1 week before surgery and 181 patients met the eligibility criteria. There were 38 males and 143 females, with 148 benign thyroid nodules (137 nodular goiters, seven follicular adenomas, and four Hashimoto’s thyroiditis) and 111 malignant nodules (107 papillary thyroid carcinomas, three follicular carcinomas, and one medullary carcinoma). The patients were 50.6 ± 14.2 (range, 12–84) years. No significant differences in patient age (*P* = 0.700) or gender (*P* = 0.419) were found between the benign and malignant nodule groups (Table 1). There were 140 nodules in the right lobe, 104 in the left lobe and 15 in the isthmus. A single nodule was found in 126 patients and multiple nodules in 55 patients. Fourteen patients had concurrent benign and malignant nodules.

**Table 1:** Characteristics of the patients and MRI features of the thyroid nodules

Parameters	Benign nodules	Malignant nodules	*P*
Age (years)	57.4 ± 13.4^b^	45.5 ± 12.7^b^	0.700
Gender (male: female)	37:111^a^	23:88^a^	0.419
Size (mm)	20.1 ± 12.6^b^	12.0 ± 7.5^b^	< 0.001
Location			0.525
Right lobe	78 (52.7%)	62 (55.9%)	
Isthmus of thyroid	7 (4.7%)	8 (7.2%)	
Left lobe	63 (42.6%)	41 (36.9%)	
Margin			0.080
Clear	45 (30.4%)	23 (20.7%)	
Vague	103 (69.6%)	88 (79.3%)	
Shape			< 0.001
Regular	136 (91.9%)	11 (9.9%)	
Irregular	12 (8.1%)	100 (90.1%)	
Pseudocapsule sign			< 0.001
Yes	70(47.3%)	6(5.4%)	
No	78(52.7%)	105(94.6%)	
Signal intensity			0.079
Homogeneous	17 (11.5%)	22 (19.8%)	
Heterogeneous	131 (88.5%)	89 (80.2%)	
Cystic degeneration			< 0.001
Yes	102(68.9%)	19(17.1%)	
No	46(31.1%)	92(82.9%)	
High signal cystic area on T1WI			< 0.001
Yes	63(42.6%)	9(8.1%)	
No	85(57.4%)	102(91.9%)	
Ring sign			< 0.001
Yes	2(1.4%)	72(64.9%)	
No	146(98.6%)	39(35.1%)	
ADC values (×10^−3^ mm^2^/s)	1.946 ± 0.349^b^	1.260 ± 0.225^b^	< 0.001
Degree of enhancement			0.012
Mild	11 (7.4%)	10 (9.0%)	
Moderate	89 (60.1%)	82 (73.9%)	
Marked	48 (32.5%)	19 (17.1%)	

Data are numbers of patients, unless indicated otherwise. Numbers in parentheses are percentages

^a^Gender ratio

^b^means ± standard deviation

### Association between MRI parameters and malignant nodules

**Fig. 2:**
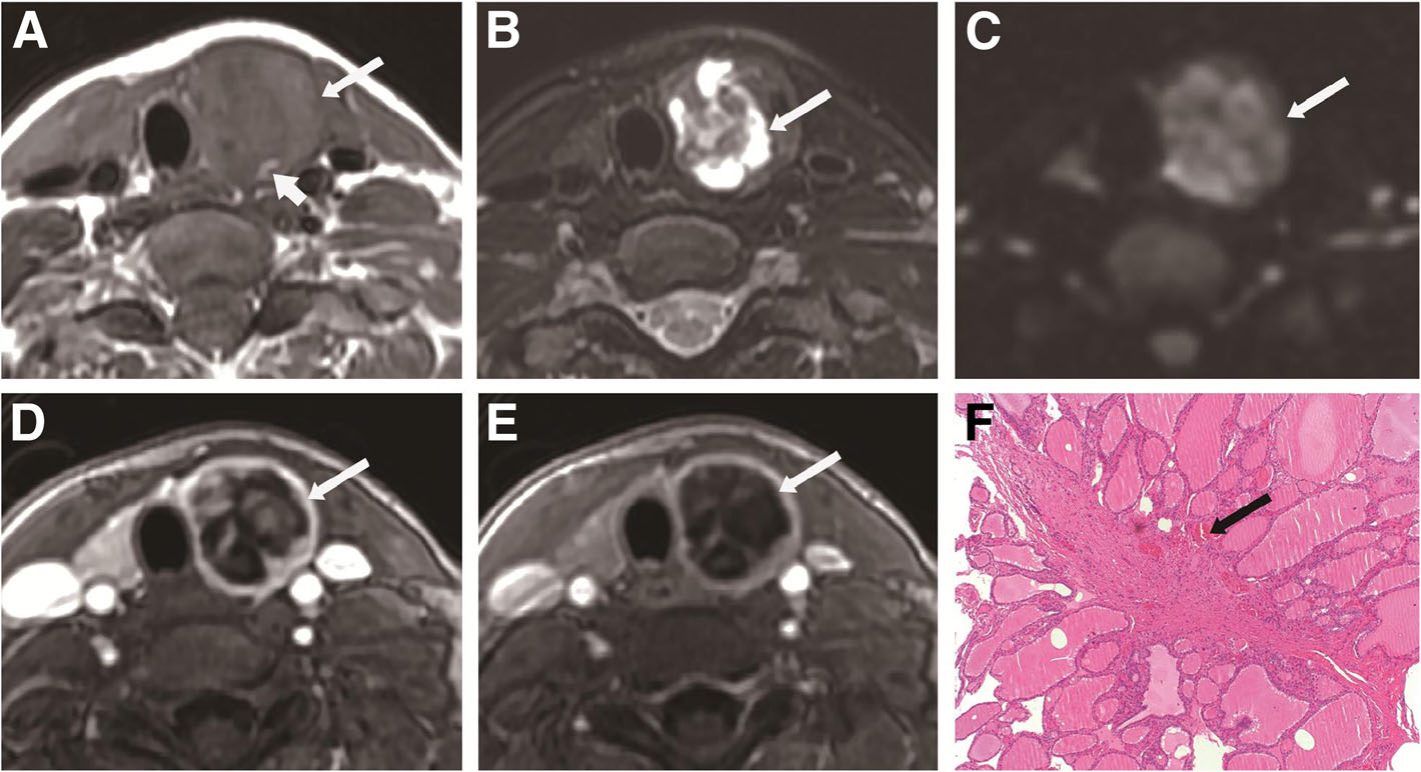
A 47-year-old woman with thyroid nodular goiter in the left thyroid lobe. **a** Axial T1-weighted image showing a heterogeneous isointense nodule (long arrowhead) with patchy hyperintense signal (white arrow) in the left lobe. **b** Axial T2-weighted image showing a heterogeneous hyperintense nodule with cystic change (white arrow) in the left lobe. **c** Axial DWI image showing a hyperintense nodule (white arrow) with ADC value of 1.990 × 10^− 3^ mm^2^/s. **d** Axial contrast-enhanced image showing a heterogeneous hyperintense lesion with regular shape and clear margin in the left thyroid lobe during early phase. **e** Axial contrast-enhanced image showing the pseudocapsule sign (white arrow) in the left thyroid lobe during delayed phase. **f** Histopathological hematoxylin and eosin (H&E, × 40) staining showing heterogeneous follicular hyperplasia with colloid and hemorrhage (white arrow)

**Fig. 3:**
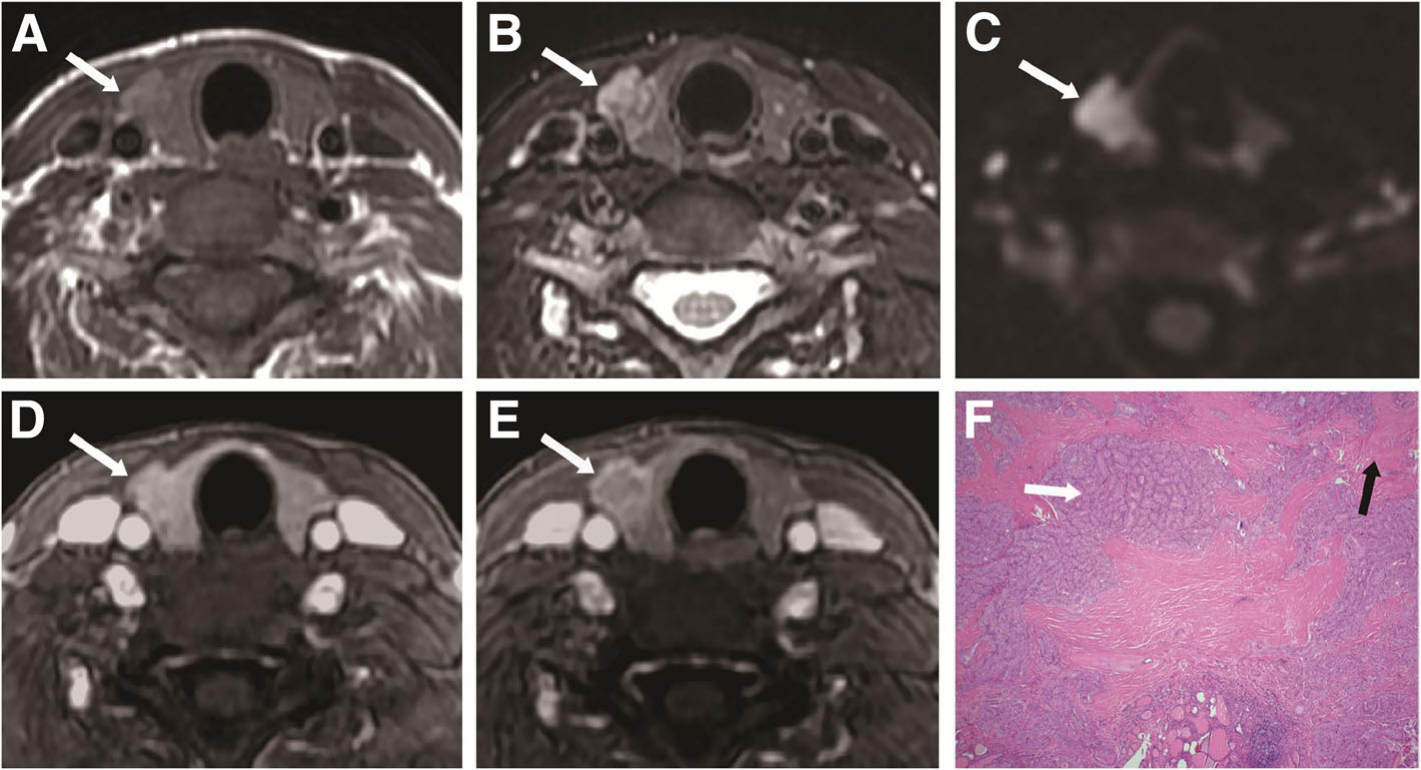
A 44-year-old woman with thyroid papillary carcinoma in the right thyroid lobe. **a** Axial T1-weighted image showing an isointense lesion (white arrow) in the right thyroid lobe. **b** Axial T2-weighted image showing a heterogeneous hyperintense lesion with extrathyroidal extension (white arrow) in the right thyroid lobe. **c** Axial DWI image showing a hyperintense nodule (white arrow) with ADC value of 1.070 × 10^− 3^ mm^2^/s. **d** Axial contrast-enhanced image showing a moderately enhanced mass-like lesion with irregular shape and extrathyroidal extension (white arrow) in the right thyroid lobe during the early phase. **e** Axial contrast-enhanced image showing central wash-out of the lesion, with the ring sign and irregular shape (white arrow) in the right thyroid lobe during the delayed phase. **f** Histopathological hematoxylin and eosin (H&E, × 40) staining showing papillary growth of cancer cells (white arrow) and dense fibrous tissue (black arrow)

**Fig. 4:**
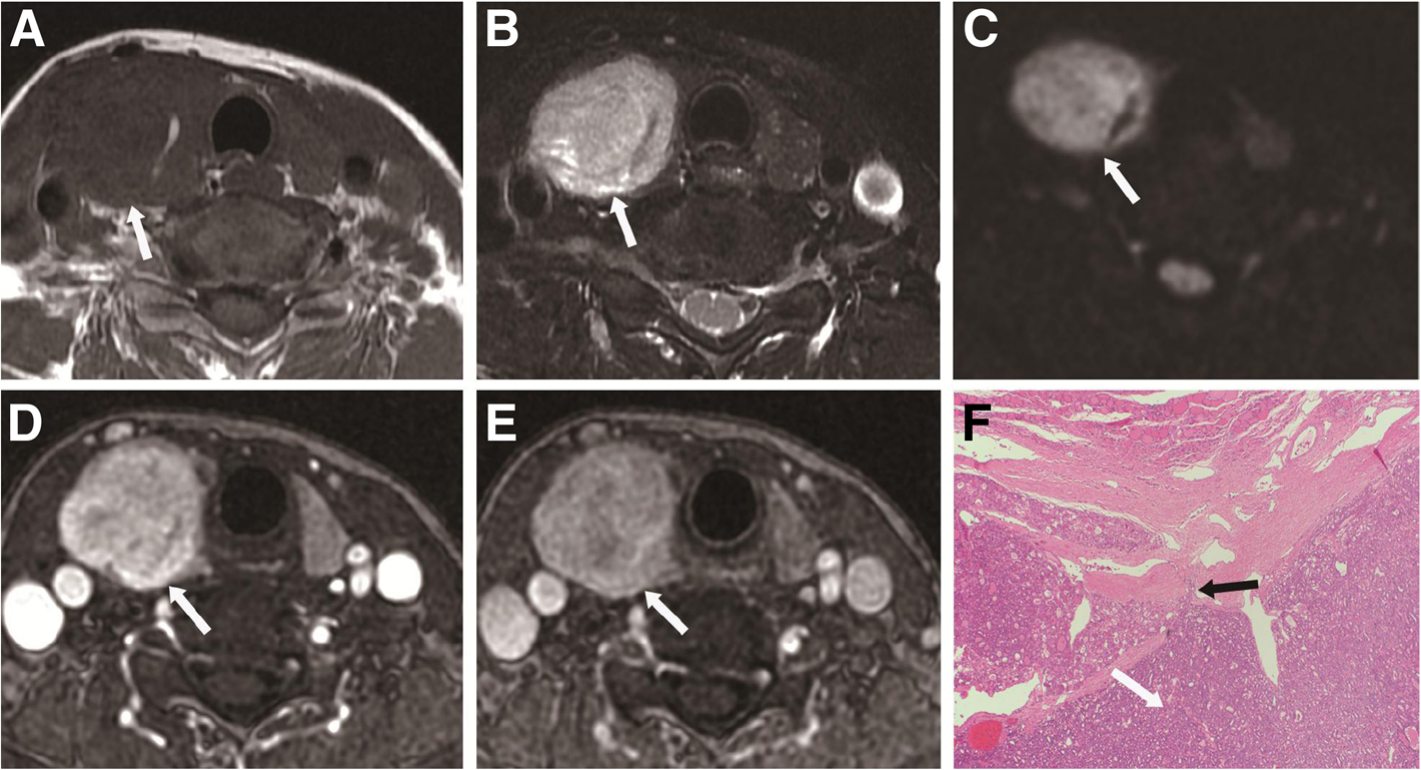
A 53-year-old man with thyroid follicular carcinoma in the right thyroid lobe. **a** Axial T1-weighted image showing a heterogeneous isointense lesion (white arrow) in the right thyroid lobe. **b** Axial T2-weighted image showing a heterogeneous hyperintense lesion with regular shape and clear margin (white arrow) in the right thyroid lobe. **c** Axial DWI image showing a markedly hyperintense nodule (white arrow) with ADC value of 0.998 × 10^− 3^ mm^2^/s. **d** Axial contrast-enhanced image showing a markedly enhanced lesion with regular shape and clear margin (white arrow) in the right thyroid lobe during early phase. **e** Axial contrast-enhanced image shows a wash-out enhanced lesion with regular shape and clear margin (white arrow) in the right thyroid lobe during delayed phase. **f** Histopathological hematoxylin and eosin (H&E, × 40) staining showing abundant follicular hyperplasia (white arrow) and tumor cell invasion in the peripheral stroma (black arrow)

Table 1 shows the univariable analyses of the association between MRI features and malignant nodules. The benign nodules (20.1 ± 12.6 mm, range: 3.8–64.6 mm) were significantly larger than the malignant thyroid nodules (12.0 ± 7.5 mm, range: 4.3–47.3 mm) (*P* < 0.001). The ADC of the benign group (1.95 ± 0.35 × 10^− 3^ mm^2^/s, range: 0.99–3.16 × 10^− 3^ mm^2^/s) was significantly higher than that of the malignant group (1.26 ± 0.23 × 10^− 3^ mm^2^/s, range: 0.77–2.22 × 10^− 3^ mm^2^/s) (*P* < 0.001). Cystic degeneration, the pseudocapsule sign, and high signal cystic area on T1WI were more common in benign thyroid nodules (all *P* < 0.001). The ring sign in the delayed phase and irregular shape after contrast agent was significantly more common in the malignant group (both *P* < 0.001) (Figs. 2, 3 and 4). There were no significant differences regarding margin and signal heterogeneity between the malignant and benign groups (both *P* > 0.05).

### Multivariable analysis

Table 2 shows the results of the final logistic regression model. The ADC value (OR = 694.006, *P* < 0.001), irregular shape (OR = 32.798, *P* < 0.001), ring sign in the delayed phase (OR = 20.381, *P* = 0.004), and cystic degeneration (OR = 8.468, *P* = 0.016) were independently associated with malignant thyroid nodules, with 0.961 accuracy.

**Table 2:** Independent Variables in the regression equation

Parameters	OR	95% CI		*P*
		Lower	Upper	
ADC	694.006	49.769	9677.615	< 0.001
Irregular shape	32.798	6.495	165.619	< 0.001
Ring sign	20.381	2.668	155.717	0.004
Cystic degeneration	8.468	1.487	48.225	0.016

### ROC curve analysis

**Fig. 5:**
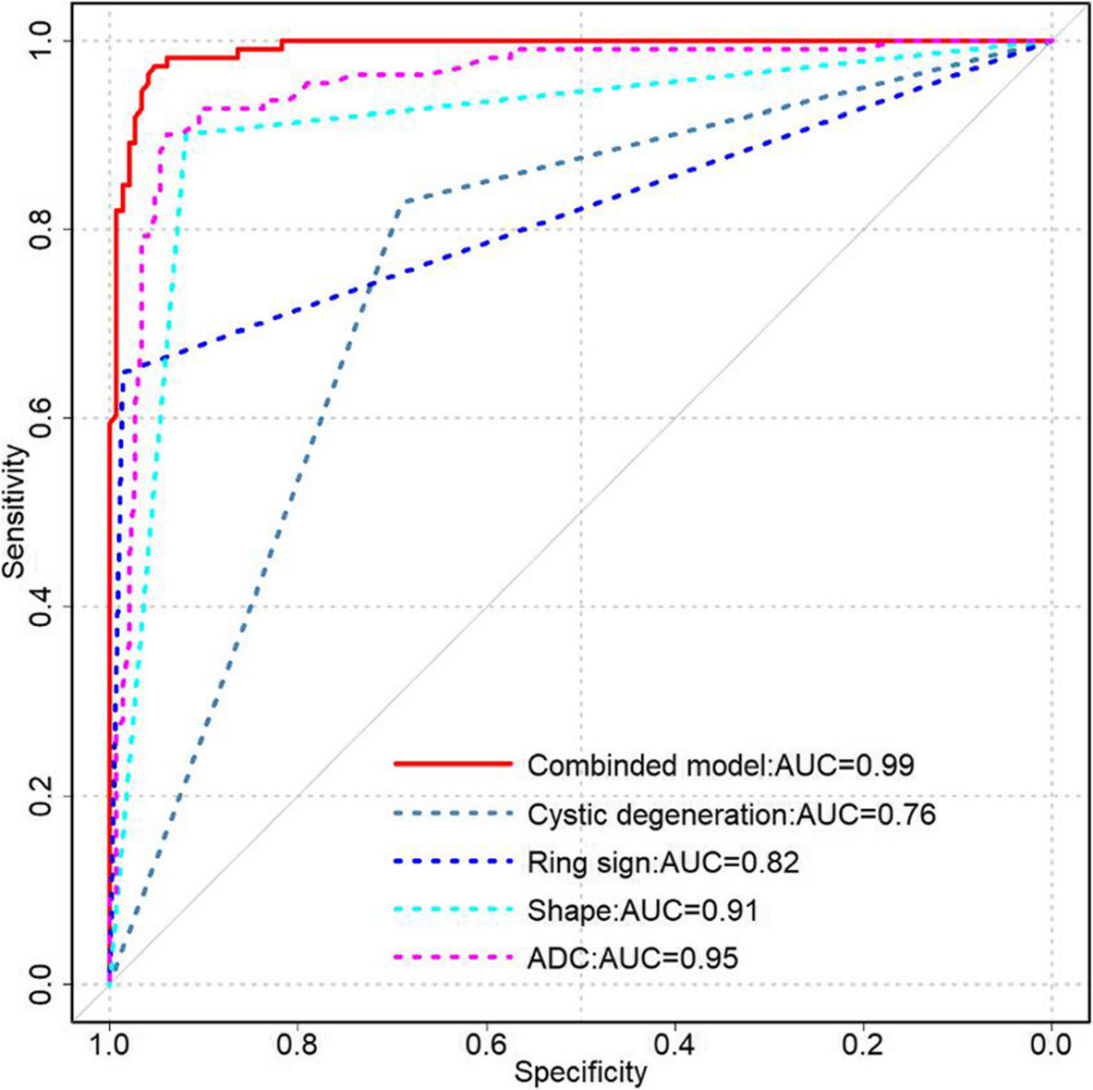
Receiver operating characteristic (ROC) curve of the apparent diffusion coefficient (ADC) values, shape, ring sign, cystic degeneration, and the combined model for differentiating benign from malignant thyroid nodules

The ROC curve analysis revealed that the best cut-off of ADC values achieved AUC of 0.95, 0.90 sensitivity and 0.91 specificity. Cystic degeneration, ring sign, and irregular shape had AUC of 0.76, 0.82, and 0.91, respectively. When the independent factors were combined, the diagnostic performance was improved to an AUC of 0.99, 0.97 sensitivity, and 0.95 specificity (Fig. 5).

## Discussion

DWI has a good diagnostic value for thyroid disease. Previous studies [17, 19] showed that ADC values of the thyroid gland can be used to assess the activity of Graves’ disease and to differentiate Graves’ disease from painless thyroiditis in patients with untreated thyrotoxicosis. The ADC value is a noninvasive imaging approach used for differentiating malignant from benign solitary thyroid nodules, but the published protocols suffer from flaws and previous studies focus on the ADC [11, 12, 14–16, 18]. Therefore, the aim of the present study was to investigate the diagnostic performance of multiple MRI parameters in differentiating malignant from benign thyroid nodules. The results showed that ADC, irregular shape, ring sign, and cystic degeneration were independently associated with malignant thyroid nodules. While the irregular shape, ring sign, and cystic degeneration can be subjective and dependent upon the radiologist’s experience, ADC can provide quantitative information to differentiate thyroid carcinoma from benign thyroid nodules. The present study suggests that combining subjective MRI features to a quantitative measurement could improve the diagnostic yield of MRI for malignant thyroid nodules.

The incidence of thyroid cancer is rapidly increasing, with a 3% estimated annual increase in the United States [20]. Similar patterns have been reported in Canada, Australia, China, and Western Europe [20, 21]. US is the primary imaging modality to assess thyroid nodules [22]. FNAB is an accurate and cost-effective method for evaluating thyroid nodules, with high diagnostic sensitivity and specificity [23]. Nevertheless, US-guided FNAB is an invasive procedure and cannot distinguish between benign and malignant non-papillary follicular and oxyphilic cell lesions [6, 12]. MRI is an effective noninvasive modality to differentiate malignant from benign tumors [24]. A number of MRI studies examined the ADC values of thyroid nodules [8, 22, 25], but image quality was considered to be relatively poor because of susceptibility artifacts, motion artifacts, and low signal-to-noise ratio with previous head and neck joint coil. One previous study showed that only 26/40 patients had images that could be interpreted because of distortion [8]. Another study showed that patient motion was the major factor of exclusion due to breathing, swallowing, and coughing [2]. In addition, the b value is a critical factor affecting image quality and ADC values. When low b value is used, the ADC value tends to be higher due to the contribution of perfusion. Applying high maximum b values may be preferable when ADC measurements are performed to differentiate malignant from benign tissues, exclusively based on their water diffusion characteristics. Nevertheless, the signal-to-noise ratio decreases as the b value increases, thus limiting the maximum b value. In addition, we used a neck surface coil to increase the signal-to-noise ratio. Indeed, because the coil was close to the surface of neck, it could minimize the air-tissue boundary for reducing susceptibility artifacts. Therefore, a relatively high b value (800 × 10^− 3^ s/mm^2^) was used, which could better reflect the actual diffusion characteristics in this study. In addition, we used special techniques to improve image quality. A relatively small FOV (14 × 14 cm) was used to reduce susceptibility artifacts. Shim blocks were used to optimize magnetic homogeneity in the thyroid region. All patients received respiratory training to improve movement-related problems. We used a breath-hold technique on dynamic contrast-enhanced MRI phase to reduce breathing motion artifacts and added saturated zone to reduce carotid artery pulsatile artifacts. Therefore, 239 of the 254 patients showed excellent image quality in this study.

Some studies have shown that DWI can differentiate benign from malignant thyroid nodules [7, 12, 14, 26]. Nevertheless, the numbers of cases included in these studies were relatively small and the findings were sometimes inconclusive [16, 26]. The sample size of the present study was relatively large, with 181 patients and 259 thyroid lesions. Malignant nodules in this study showed lower ADC values compared with benign nodules. Logistic regression showed that ADC values had a high prediction value for the malignant status of thyroid lesions. Cytological features of malignant thyroid nodules in this study included enlarged and irregular nuclei, increased cell density, and relatively severe desmoplastic response, whereas abundant follicles, extracellular fluid and smaller cell density resulted in higher ADC values in adenoma and nodular goiter. These results were consistent with previous studies [18, 26].

Dynamic contrast enhancement can play a complementary role in the diagnosis of thyroid carcinoma. During the delayed phase, the ring sign (with a central washout enhancement) was seen in a large number of malignant thyroid nodules, which was not reported in previous studies. The central tumor area with washout indicates active growth of tumor cells, whereas the peripheral area is mainly composed of loose connective tissue with abundant intercellular matrix. Peripherally enhanced areas in malignant thyroid tumors during the delayed phase may also be related to the fibrous stroma of the tumor and the presence of vascular fibrotic stroma. Malignant thyroid nodules in the present study showed irregular shape after contrast agent. The histopathological characteristics of thyroid carcinoma indicate the invasive and heterogeneous growing pattern. One recently published study [27] also showed that irregular margins on US were strong predictor of malignancy.

In the present study, cystic degeneration, high signal cystic area on T1WI, and the pseudocapsule sign were significantly more frequent in benign thyroid nodules than in malignant nodules. Nodular goiter was the main pathological type of benign thyroid nodules in this study. Due to the relative abundance of colloid follicles and hemorrhage, nodular goiter showed cystic changes and high signal intensity in cystic areas. Shi et al. [26] showed similar results. Na et al. [28] showed that the risk of malignancy of partially cystic nodules was lower than the risk of malignancy of purely solid nodules. Similar to previous studies [28], the present study showed that 68.9% of thyroid benign nodules showed cystic changes, but only 17.1% of the malignant lesions showed cystic changes. The pseudocapsule sign was not the real capsule of tumor, but showed a clear capsule after contrast agent administration because the tumor compressed the peripheral thyroid parenchyma and caused fibrosis. Therefore, the pseudocapsule sign indicates a chronic and benign pathological process.

In this study, the enhancement degree between the two groups was significantly different, but there was major overlap between the two groups. Nodular goiter and adenoma showed moderate or marked enhancement with abundant hyperplasia of thyroid follicles. Follicular thyroid carcinoma showed marked enhancement because of abundant hyperplasia of thyroid follicles and neovascularity. Papillary thyroid carcinomas demonstrated moderate or marked enhancement with increased cell density, severe desmoplastic response, and cell proliferation, which were consistent with previous studies [26, 29].

Taken together, the present study strongly suggests that multiple MRI parameters should be considered when evaluating thyroid nodules. While irregular shape, ring sign, and cystic degeneration can be subjective and dependent upon the radiologist’s experience, ADC can provide quantitative information to differentiate thyroid carcinoma from benign thyroid nodules. On the other hand, whether the parameters observed in the present study are better than other modalities such as US, computed tomography, and scintigraphy [30] require additional studies.

This study has several limitations. Firstly, this study was retrospective in design, leading to selection bias and therefore undermining the validity of the results. Prospective studies with larger sample size would increase the credibility of the results. Secondly, thyroid nodules measuring < 3 mm were not included. Improvements in MRI software and using smaller slice gaps may facilitate the detection of smaller lesions in future studies. Importantly, it has been shown that small thyroid lesions are at higher risk of malignancy than larger ones [21]. Therefore, the present study probably underreported the number of malignant lesions. Thirdly, in this study, the major malignant pathological type was papillary carcinoma, while the major benign type was nodular goiter, similar to previous studies [18, 26]. Nevertheless, the underrepresentation of rarer pathological types could bias the results. We need to enlarge the samples in the following studies. Fourthly, our center only has a 1.5-T MRI scanner and differences in imaging parameters for malignant thyroid nodules could not be compared with a 3-T scanner. In addition, 1.5 T MRI scanners cannot implement multiple b values and we applied a high b value to better reflect the value of diffusion. Meanwhile, with technological and software development, some advanced diffusion imaging like diffusion tensor imaging has been used for the differentiation between malignant and benign tumors of the head and neck [31]. Our center could not implement those different advanced diffusion imaging modules. Furthermore, we could not determine the K-trans value because dynamic contrast enhancement at our center is routinely done at 30 s, 60 s, and then every minute, and only the trend of dynamic enhancement could be extracted. Fifthly, we used a neck surface coil to increase signal noise ratio and many techniques to reduce artifacts, but we did not compare the differences among the coils and techniques. In addition, we did not compare the difference among different pathological types. Finally, the irregular shape, ring sign, and cystic degeneration are indeed subjective, but these parameters had nevertheless high sensitivity and specificity. These parameters have not been reported before, and could have some value for the management of patients with thyroid nodule.

## Conclusions

Multiple MRI parameters could be helpful to differentiate malignant thyroid nodules from benign nodules. The logistic regression showed that ADC value could discriminate between benign and malignant thyroid nodules with a good performance. Subjective signs such as the ring sign, irregular shape, and cystic degeneration associated with malignant thyroid nodules could provide complementary information for differentiation. Combining subjective MRI features to a quantitative measurement could improve the diagnostic performance of MRI for malignant thyroid nodules.

## Abbreviations

ADC: Apparent diffusion coefficient
AUC: Area under the curve
DWI: Diffusion weighted imaging
FNAB: Fine-needle aspiration biopsy
FNAC: Fine-needle aspiration cytology
FOV: Field of view
NEX: Number of excitations
ROC: Receiver operator characteristic
ROI: Region of interest
T1WI: T1-Weighted imaging
T2WI: T2-weighted images
TE: Echo time
TI-RADS: Thyroid Imaging Reporting and Data System
TR: Repetition time
US: Ultrasound
